# Supplementary material for: Identification of photoperiod-induced specific miRNAs in the adrenal glands of Sunite sheep (Ovis aries)
Source: Front Vet Sci. 2022 Jul 22;9:888207. doi: 10.3389/fvets.2022.888207 (PMC9354845; doi:10.3389/fvets.2022.888207)
Supplement: Supplementary file 1 [file Data_Sheet_1.ZIP › Supplementary file/miRDeep2 file.zip!/miRDeep2 file/Novel_Structure/SPLP42A1/Novel_621.pdf]

Provisional ID	:	NC_019471.2_20504
Score total	:	162.2
Score for star read(s)	:	3.9
Score for read counts	:	154.1
Score for mfe	:	2.6
Score for randfold	:	1.6
Score for cons. seed	:	
Total read count	:	314
Mature read count	:	305
Loop read count	:	0
Star read count	:	9

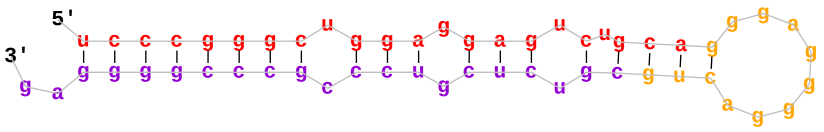

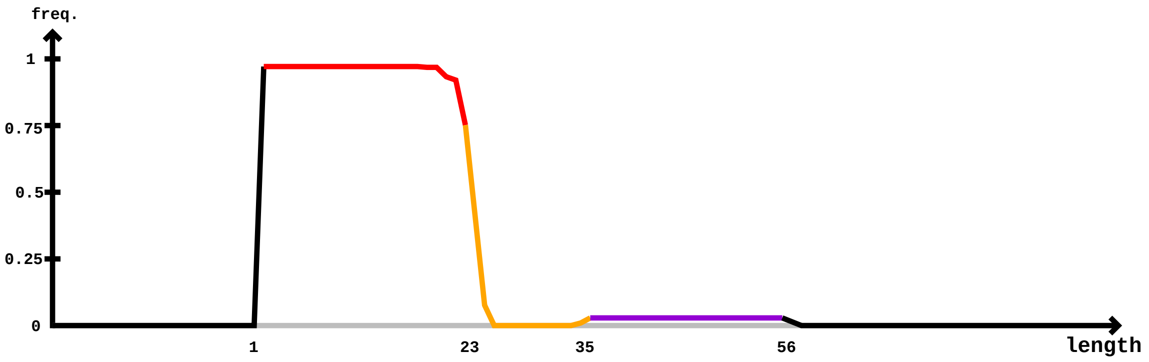

Mature Star

```
5'- ggaccugggcaggggcgggguccccgggcuggaggagucugcagggaggggacugcgucucgucccgcccggggagaccgccccuugccgcucccaggacgguggauagc -3'  obs
    ggaccugggcaggggcgggguccccgggcuggaggagucugcagggaggggacugcgucucgucccgcccggggagaccgccccuugccgcucccaggacgguggauagc      exp
    .......(((((((((((..((((((((.(((.(((.(.((((........))))).))).))).))))))))...))))))).))))(((.(((......)))..)))  reads  mm  sample
    ....................ucccgggcuggaggagu........................................................................  1      0   seq
    ....................ucccgggcuggaggagucu......................................................................  11     0   seq
    ....................ucccgggcuggaggagucug.....................................................................  4      0   seq
    ....................ucccgggcuggaggagucugc....................................................................  53     0   seq
    ....................ucccgggcuggaggagucugca...................................................................  145    0   seq
    ....................ucccgggcuggaggagucugcag..................................................................  67     0   seq
    ....................ucccgggcuggaggagucugcagg.................................................................  24     0   seq
    .....................................................gcgucucgucccgcccggggaga.................................  3      0   seq
    ......................................................cgucucgucccgcccggggag..................................  6      0   seq
```
